# The LIFEwithIBD Intervention: Study Protocol for a Randomized Controlled Trial of a Face-to-Face Acceptance and Commitment Therapy and Compassion-Based Intervention Tailored to People With Inflammatory Bowel Disease

**DOI:** 10.3389/fpsyt.2021.699367

**Published:** 2021-08-19

**Authors:** Inês A. Trindade, Joana Pereira, Ana Galhardo, Nuno B. Ferreira, Paola Lucena-Santos, Sérgio A. Carvalho, Sara Oliveira, David Skvarc, Bárbara S. Rocha, Francisco Portela, Cláudia Ferreira

**Affiliations:** ^1^Faculty of Psychology and Education Sciences, CINEICC, University of Coimbra, Coimbra, Portugal; ^2^Department of Molecular and Clinical Medicine, Institute of Medicine, University of Gothenburg, Sahlgrenska Academy, Gothenburg, Sweden; ^3^Instituto Superior Miguel Torga, Coimbra, Portugal; ^4^School of Social Sciences, University of Nicosia, Nicosia, Cyprus; ^5^Universidade Lusófona de Humanidades e Tecnologias, Escola de Psicologia e Ciências da Vida, HEI-Lab, Lisbon, Portugal; ^6^School of Psychology, Deakin University, Geelong, VIC, Australia; ^7^Faculty of Pharmacy, University of Coimbra, Coimbra, Portugal; ^8^Gastroenterology Service, Centro Hospitalar e Universitário de Coimbra (CHUC), Coimbra, Portugal

**Keywords:** acceptance and commitment therapy, compassion, inflammatory bowel disease, mindfulness, randomized controlled trial, study protocol

## Abstract

**Background:** There is ample evidence of the high mental health burden caused by Inflammatory Bowel Disease (IBD). Several constructs such as experiential avoidance, cognitive fusion, shame, and self-criticism have recently emerged as potential intervention targets to improve mental health in IBD. Psychotherapeutic models such as Acceptance and Commitment Therapy and compassion-based interventions are known to target these constructs. In this protocol, we aim to describe a two-arm Randomized Controlled Trial (RCT) testing the efficacy of an ACT and compassion-focused intervention named Living with Intention, Fullness, and Engagement with Inflammatory Bowel Disease (LIFEwithIBD) intervention + Treatment As Usual (TAU) vs. TAU in improving psychological distress, quality of life, work and social functioning, IBD symptom perception, illness-related shame, psychological flexibility, self-compassion, disease activity, inflammation biomarkers, and gut microbiota diversity.

**Methods:** This trial is registered at ClinicalTrials.gov (Identifier: NCT03840707, date assigned 13/02/2019). The LIFEwithIBD intervention is an adaptation to the IBD population of the Mind programme for people with cancer, an acceptance, mindfulness, and compassion-based intervention designed to be delivered in a group format. The LIFEwithIBD intervention's structure and topics are presented in this protocol. Participants were recruited at the Gastroenterology Service of the Coimbra University Hospital between June and September 2019. Of the 355 patients screened, 61 participants were selected, randomly assigned to one of two conditions [experimental group (LIFEwithIBD + TAU) or control group (TAU)] and completed the baseline assessment. Outcome measurement took place at baseline, post-intervention, 3- and 12-month follow-ups.

**Discussion:** Results from this RCT will support future studies testing the LIFEwithIBD intervention or other acceptance and/or compassion-based interventions for IBD.

## Introduction

Inflammatory bowel disease (IBD) is a group of chronic conditions comprised mainly of Crohn's disease and ulcerative colitis and characterized by inflammation of the intestinal tract, usually leading to symptomatology such as diarrhea, abdominal pain, rectal bleeding, fatigue, and weight loss. IBD comprises periods of active disease, with increased symptomatology, alternating with periods of remission, in a usually unpredictable manner (1). People living with IBD are faced with significant disease burden (2–4), with impaired daily functioning (5), and are at risk of developing anxiety and depression disorders (6, 7). During periods in which symptoms are present (active disease), the rates of anxiety and depressive disorders can go up to 66.4 and 34.7%, respectively (7). During remission, rates are lower but still substantial (anxiety: 28.2%; depression: 19.9%), contrasting with rates presented by individuals from the general population (anxiety: 9.6%; depression: 13.4%) (7). Furthermore, depression contributes to disease activity, being associated with more aggressive IBD symptomatology (8). This effect is produced through depression's association with poor disease self-management and increased immune system activation (9, 10). Increased inflammation and disease symptomatology, in turn, increase depression levels (9). However, the overlap and bidirectionality between IBD and depression is still vaguely considered in IBD treatment, and depression has been reported to be underrecognized and undertreated in IBD (9, 11).

Findings from recent studies examining which factors are linked to poor mental health outcomes in people with IBD have suggested that this population may benefit from psychological interventions focused on promoting acceptance and cultivating self-compassion (12–15). Arguments for interventions based on psychological flexibility lie on studies showing the detrimental effects of experiential avoidance (i.e., unwillingness to have internal experiences such as thoughts, emotions, and physical sensations, consequently leading to attempts to avoid them) (16) and cognitive fusion (entanglement with thoughts which leads to taking them as literal truths) (17), on mental health and quality of life outcomes. In people with IBD, cognitive fusion was shown to longitudinally influence changes on perceived physical and psychological well-being for 18 months, over and above IBD symptomatology's influence (13), and to longitudinally predict depression symptoms (18). Additionally, fusion with distressing IBD-related thoughts is linked with lower psychological health and may explain the effects of painful experiences such as shame on this outcome (14). Experiential avoidance, in turn, seems to mediate the effects of IBD symptomatology on well-being and mental health indicators (19, 20). Altogether, these studies indicate that fusion with negative thoughts and attempts at controlling or avoiding internal experiences appear to increase the suffering experienced by people with IBD and potentially contribute to the effects of painful IBD-related experiences on psychological distress. Therefore, the testing of psychological approaches aiming at improving psychological flexibility for better living with this condition is of paramount relevance.

Acceptance and Commitment Therapy [ACT] (16) uses acceptance, defusion, and mindfulness processes, allied with commitment and behavior change techniques, to produce greater psychological flexibility. ACT has been tested in several contexts for the past two decades, with current empirical evidence showing that it is generally superior to inactive controls, treatment as usual (TAU), and most active intervention conditions for anxiety, depression, substance use, pain, and transdiagnostic groups (21). A systematic review examining ACT's use in chronic medical conditions found, nonetheless, that although ACT shows promise, it is not yet a well-established intervention for these populations, recommending that more trials should be conducted (22). The emerging evidence of ACT in addressing symptoms and mental health issues in gastrointestinal disorders has been highlighted (23), and ACT is regarded by people with IBD and health professionals as a helpful approach, especially shortly after diagnosis (24). Nevertheless, to our current knowledge, only one Randomized Controlled Trial (RCT) has been performed in the IBD population. This study showed that an 8-week ACT intervention was superior to TAU in improving stress, depression, and general well-being (25). Nonetheless, several mindfulness-based interventions have been tested in IBD, with results showing efficacy in improving symptoms management (26), fatigue, psychosocial functioning (27), and inflammatory biomarkers (28). Although ACT incorporates mindfulness as a core element in promoting contact with the present moment, it does so in a strictly behavioral approach (29), rather than in its entire philosophical and ethical dimensions (30), and it also includes other techniques for defusion, acceptance, and for promoting values-based action.

Recently, a few studies have called for the pertinence of adding compassion-based components to such interventions based on the fact that people with IBD are impacted by stigma, shame, and self-criticism (12, 15, 31). Concerns of people with IBD can include being treated as different, being ostracized by society, or feeling a burden to others (32, 33). IBD characteristics such as the gastrointestinal nature of symptoms, the chronic and relapsing disease course, and concealability, as well as the historical view of it being a psychosomatic condition make IBD prone to stigmatization (34, 35). The stigmatization of IBD, in turn, is known to impact quality of life, psychological functioning, and treatment adherence (12). Illness-related shame in people with IBD also seems to negatively influence psychological health and quality of social relationships (31). People with IBD may be self-critical, perceiving themselves as inadequate, flawed, or defective, which seems to exacerbate the effects of IBD symptom perception and illness shame on depressive symptomatology (15).

The efficacy of interventions that incorporate compassion cultivation in decreasing psychological distress and psychopathological symptoms, and improving quality of life and well-being, has gained growing evidence (36). Developing self-compassion (i.e., sensitivity to personal suffering and a motivation to alleviate and prevent it in a self-kind and courageous manner) (37) seems to be especially useful to tackle shame and self-criticism (38). Self-compassion is operated through different brain systems than self-criticism (39). It is suggested to be rooted in a soothing-affiliative affect regulation system that counteracts threat-focused emotional responses and cognitive patterns, such as depression, anxiety, shame, and self-criticism (40). In line with this, a recent longitudinal study in people with IBD showed self-compassion to be a protective factor to the experience of stress, depressive symptoms, and anxiety (41). Interventions that promote self-compassion are feasible (42) and present promising results in the context of chronic illnesses, such as persistent pain (43), acquired brain injury (44), and chronic skin conditions (45). Although these data seem to indicate that helping cultivate self-compassion may be useful in IBD, no compassion-based intervention has yet been tested in this population.

The integration of self-compassion components in ACT and mindfulness-based interventions has been an emerging research topic during the past decade. These approaches are considered to be compatible and complementary (46–48). Their integration may have added benefits and appears to be feasible and effective in improving quality of life, experiential avoidance, shame, or self-criticism in populations such as men with HIV (49), women with binge eating (BEfree intervention) (50), obesity (Kg-Free intervention) (51), and breast cancer (Mind programme) (52).

Taking into consideration the high mental health burden of IBD, the pertinence of targeting experiential avoidance, cognitive fusion, shame, and self-criticism in this population, and the feasibility of integrating ACT and compassion-based interventions, it is the aim of the current project to test the efficacy of such integrative intervention tailored to IBD.

### Aims of this Study

This study aims to conduct a two-arm RCT to test the efficacy of a new integrative ACT and compassion-based intervention, the Living with Intention, Fullness, and Engagement with Inflammatory Bowel Disease (LIFEwithIBD) intervention, which was adapted from the Mind programme for people with cancer (52) to the IBD context. This intervention (LIFEwithIBD + TAU)'s superiority in improving psychological distress (primary outcome), quality of life, work and social functioning, IBD symptom perception, illness-related shame, psychological flexibility, self-compassion, disease activity, inflammation biomarkers, and gut microbiota diversity will be compared to TAU.

The contents of the LIFEwithIBD intervention and the design of the RCT are presented in this paper.

## Methods and Analysis

This trial is registered at ClinicalTrials.gov (Identifier: NCT03840707, date assigned 13/02/2019). The planning and implementation were carried out per ethical recommendations outlined by the American Psychological Association (53) and the World Medical Association's Declaration of Helsinki (54). Authorization for sample recruitment was obtained from the Portuguese Data Protection Authority (reference number 3461/2017, 2017-03-24) and the Research Ethics Committees of the Coimbra Hospital and Universitary Center (CHUC).

### Participant Recruitment

Participants were recruited among outpatients from the IBD clinic of the Gastroenterology Service of the CHUC between June and September 2019. Patients were invited to participate in the study by their IBD physician during a scheduled appointment, after assessment by the physician of exclusion criteria (a) (see below). Then, patients were screened by a psychologist through a short interview (to present the project, obtain informed consent, and assess eligibility).

#### Participant Selection

Inclusion criteria were: (a) 18 to 65 years old; (b) being able to read and write Portuguese; (c) having an IBD diagnosis for at least 6 months; (d) being able to give informed consent. Exclusion criteria were: (a) having started a new treatment for IBD in the previous 6 months (in the case of anti-TNF and immunosuppressive therapy) or 2 months (in the case of steroid or aminosalicylate therapy); (b) presenting a diagnosed psychiatric disorder (major depressive disorder, psychotic disorder, bipolar disorder, substance abuse), severe depression, or suicidal ideation (assessed by the Patient Health Questionnaire-9 [PHQ-9 (55)])^1^; (c) undergoing any other form of psychological intervention; (d) being pregnant. Patients non-eligible due to criteria (b) were referred to national mental healthcare services.

### Sample Size

An *a priori* power analysis for this study was performed. In the absence of meta-analytic evidence, we have used data from Wynne et al. (25). Large reductions in mood symptoms were reported at follow-up for depression [*SMD* = 0.88 (0.557–1.21)], anxiety [*SMD* = 0.75 (0.423–1.068)], and stress [*SMD* = 1.1 (0.88–1.559)], even after adjustment for baseline values. Using the smallest of these effect estimates (anxiety), the inclusion of the baseline values as a covariate, an alpha of 0.05 and 80% power, we estimate that a total sample size of *n* = 58 would be required to power the primary analysis.

### Randomization of Participants

**Figure 1:**
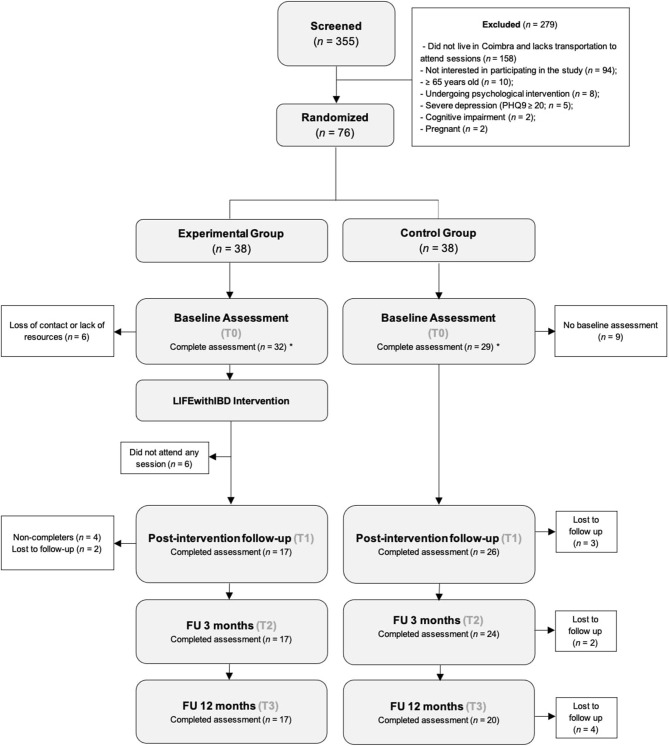
Diagram of participant enrolment. ^*^included in Intent-to-treat Analyses.

Figure 1 shows the flow of participants through the study. Of the 355 patients screened, 279 were excluded for not meeting criteria to participate. Seventy-six participants were randomly assigned to one of two conditions: experimental group (Treatment as Usual + LIFEwithIBD intervention: *n* = 38) or control group (TAU: *n* = 38), through computed-based randomization (www.random.org/lists/). Before the start of the intervention, all participants were contacted by phone. In this process, 6 patients self-excluded from the study due to a lack of resources to attend the sessions (e.g., lack of time, transportation).

### The LIFEwithIBD Intervention

The LIFEwithIBD intervention is an adaptation of the Mind programme for people with cancer (52), an acceptance, mindfulness, and compassion-based intervention designed to be delivered in a group format, with 8 weekly sessions. The Mind programme was adapted by the psychology members of the current research team for the IBD context, considering the psychological literature in this population. The results and experience derived from the Mind's pilot study and preliminary results (52) were also considered. An extra session regarding educational aspects of IBD was added, modules on shame and self-criticism were further developed, and a gratitude module replaced the forgiveness module of the Mind programme. The module on ACT's observing self was also removed, and some compassion approaches (e.g., Compassionate Image exercise) were replaced by exercises from the Mindful Self-Compassion programme (56).

The LIFEwithIBD is a manualized, group-based intervention comprising 9 weekly sessions, of about 2 h each, to be delivered to people with IBD by certified therapists with clinical experience in ACT and compassion-based interventions. Between-session assignments (usually involving meditative practices with audio guides recorded for this intervention), to be individually performed by participants ideally on a daily basis, are also included in the intervention.

Overall, the LIFEwithIBD intervention focuses on ACT topics such as acceptance of internal experiences, willingness, values, and committed action (16), as well as on mindfulness practice (57), explicit compassion-based approaches [such as Mindful Self-Compassion (56), Compassion-Focused Therapy (58), and Loving Kindness meditation (Buddhist mettā meditation) (59)], and gratitude (especially toward one's body) (59). The intervention also includes educational aspects on IBD and fatigue. In each session, participants are invited to complete experiential exercises, mindfulness and compassion meditation practices, and to discuss in small groups to share their experiences. The LIFEwithIBD first session is intended to present the intervention's aims and structure, allow participants to introduce themselves, have a sense of common areas of suffering, foster creative hopelessness, and offer mindfulness as a different way of facing difficulties. In this session, the Participants' Manual encompassing information to complement the sessions and exercises is provided to each participant. The following sessions, although focusing on different topics, have a similar structure: each session begins with a scripted meditation (some of the scripts adapted to embrace IBD specificities), a review of participants' between-session assignments (whether they encountered obstacles to the practices/exercises, what their experience was like, whether they have discovered something new, etc.), an introduction to the session's topics and in-session experiential exercises (real-life examples and metaphors are designed to facilitate the participants' practice and reinforcement of engaging in the skill addressed in the session), and ends with a mindfulness or compassion meditation practice. The between-session assignments align with the topics explored in each session and include informal practices aiming to integrate mindful awareness in everyday activities. The last session encompasses an extension of committed action (already covered in session 5), focusing on overcoming obstacles, and provides a wrap-up of the intervention, highlighting the role of psychological processes. An overview of the intervention is presented in Table 1.

**Table 1:** Overview of the LIFEwithIBD intervention.

**Session**	**Topics**	**In-session exercises/metaphors**	**Between session assignments**
1: Introduction to the programme and promotion of creative hopelessness	- Presentation of the general aims and structure of the intervention - Identification of areas of suffering and promotion of creative hopelessness - Mindfulness as an alternative strategy to deal with difficulties - The science backing mindfulness	- Introduction to mindfulness: Eating a raisin	- Informal mindfulness
2: Education about inflammatory bowel disease	- Education about inflammatory bowel disease (exploring myths, and common fears and worries) - Introduction to mindful breathing	- 3-min breathing space - Mindful Breathing	- Informal mindfulness - 3-min breathing space - Mindful Breathing
3: Body	- How our evolutionary past influences our mind - The mind-body connection - Physiological responses to emotions - Promotion of awareness of physical sensations	- Notice 5 things - Body Scan	- Informal mindfulness - Body Scan
4: Values clarification	- Clarification and definition of life values - Individual assessment of the alignment between behavior and values' importance	- 80th birthday party (values clarification) - Bull's eye exercise - Passengers on the bus metaphor - Mindfulness of sounds and thoughts	- Informal mindfulness - Mindfulness of sounds and thoughts - Getting in touch with an old friend
5: Committed action and Self-care	- Identification of objectives and obstacles to committed action - Self-care: why it is essential and how to engage in it - Education on fatigue: strategies to cope with different types of fatigue (physical and mental fatigue)	- The spoon theory - 3-min breathing space	- Informal mindfulness - 3-min breathing space - Listing of possible self-care actions
6: Compassion	- Psycho-education on shame and self-criticism - IBD as an invisible disease and IBD-related stigma - Introduction to compassion - Compassion as a strategy to manage difficult feelings and general suffering; Tacking self-criticism with self-kindness - Self-compassion and relaxation training	- Soothing rhythm breathing (58) - Loving-Kindness meditation^a^ (compassion is given to a person we love, an acquaintance, a person with whom we have minor issues, and all people with IBD)	- Informal mindfulness - Soothing rhythm breathing and Loving-Kindness
7: Acceptance and Cognitive defusion	- The power of thinking - Promotion of cognitive defusion - Promotion of acceptance and willingness through cognitive defusion	- Mindfulness practice (body, sounds and breath) - “I am… I am having the thought that I am…” exercise - Exercise “Inviting a difficulty”	- Informal mindfulness - Try on somewhat uncomfortable activities (e.g., go to the cinema without choosing the movie, sit in a different chair) to notice and embrace the feelings of discomfort that arise - Mindfulness of sounds and thoughts and/or Body Scan
8: Compassion and gratitude toward the body	- Continuation of compassionate training - The importance of gratitude for mental health - Promotion of compassion and gratitude toward our body	- Loving Kindness^a^ - Gratitude Meditation^a^ (59) - Supportive touch (56) - 3-min breathing space	- Informal mindfulness - “Ten fingers of gratitude” (60) - Compassionate Body Scan (56)
9: Committed action (continuation)/Intervention summary	- Engaging in committed action: Strategies to overcome expected obstacles - Summary of the intervention: How the promoted psychological processes are connected are interact with each other	- Obstacles in the river exercise (61) - The gardening metaphor	

*Every session starts and ends with a contemplative exercise, except for session. Exercises added in the LIFEwithIBD adaptation are cited. The remaining exercises' citations can be found in the Mind programme's article (52)*.

a *Exercises adapted to the inflammatory bowel disease's context (e.g., with added emphasis in gastrointestinal symptomatology, or on the experience of having an illness)*.

### Intervention Implementation

The LIFEwithIBD intervention was delivered to the experimental group (*n* = 32) through three groups of 11, 12, and 9 participants. Two facilitators delivered the intervention to each group, presenting the content, leading experiential exercises, and facilitating the topics' discussion. The facilitators informed the participants that they could get in contact between the sessions in case they had any queries about the sessions' contents, as recommended by the British Psychological Society (62). Facilitators highlighted the importance of at-home practice, which was reinforced by weekly email reminders. Each week, participants provided a subjective evaluation of each session (e.g., usefulness; the importance of the topic) and the frequency of at-home practice. Participants with three consecutive absences or who attended less than two-thirds of the intervention were considered non-completers. Simultaneously, all participants also received TAU [medical treatment for IBD as recommended by the European Crohn's and Colitis Organisation's guidelines (63), which can include pharmacological and/or surgical treatment].

### Treatment Integrity

Throughout the delivery of the intervention, several aspects were considered to assure that treatment integrity was maintained. All therapists had a minimum MSc degree in Clinical Psychology and were certified therapists with clinical experience in ACT and compassion-based interventions. A manual for the intervention therapists was developed, and all therapists were trained in delivering the intervention.

### Primary and Secondary Outcomes in the RCT

Participants completed self-report measures at four different times: baseline (T0), follow-up (T1), and 3-month (T2), and 12-month (T3) follow-ups (Table 2). Participants additionally provided blood and stool samples at T0 and T3. Mayo Scores or Harvey-Bradshaw Scores were assessed at T0 and T2.

**Table 2:** Schedule of enrollment, intervention, and assessments.

	**Study Period**					
	**Enrollment**	**Allocation**	**Post-allocation**			
**Timepoint**	–t2	–t1	t0	t1	t2	t3
**Enrollment**						
Informed consent	x					
Eligibility screen	x					
Blinded randomization		x				
Allocation		x				
**Intervention**						
LIFEwithIBD			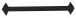			
Waiting list						
**Primary outcome**						
DASS-21			x	x	x	x
**Key mediators**						
SELFCS			x	x	x	x
CompACT			x	x	x	x
**Secondary outcomes**						
WSAS			x	x	x	x
EUROHIS-QOL-8			x	x	x	x
IBDQ-UK			x	x	x	x
CISS			x	x	x	x
IBD symptom perception			x	x	x	x
Mayo score			x		x	
Harvey-Bradshaw score			x		x	
C-Reactive protein			x			x
Hemogram			x			x
Albumin			x			x
Fecal calprotectin			x			x
Gut microbiota			x			x
Acceptability questions^a^				x		

a *only for the experimental condition*.

*DASS-21, Depression Anxiety Stress Scales; SCS, Self-Compassion Scale; CompACT, Comprehensive assessment of Acceptance and Commitment Therapy processes; WSAS, Work and Social Adjustment Scale; EUROHIS-QOL-8, EUROHIS-QOL 8-item index; IBDQ-UK, Inflammatory Bowel Disease Questionnaire–UK version; CISS, Chronic Illness-related Shame Scale; IBD Symptom Perception, measured by the IBD symptoms scale*.

#### Primary Outcome

##### Psychological distress

This outcome was assessed by the *Depression Anxiety Stress Scales* (DASS-21) (64, 65), a well-known 21-item measure of depressive (e.g., “I felt I wasn't worth much as a person”), anxiety (e.g., “I felt scared without any good reason”), and stress (e.g., “I found it hard to wind down”) symptoms during the precedent week, using a 4-point scale [ranging from “*did not apply to me at all” (0)* to “*applied to me very much, or most of the time” (3)*]. Higher scores indicate higher psychological distress. The DASS-21 presented good internal consistencies for all subscales both in original (α_depression_ = 0.88; α_anxiety_ = 0.82; α_stress_ = 0.90) and in the Portuguese psychometric validation studies (α_depression_ = 0.85; α_anxiety_ = 0.74; α_stress_ = 0.81).

#### Key Mediators

##### Self-compassion

Self-compassion was measured by the *Self-Compassion Scale* (SCS) (66, 67), a widely used 26-item measure that takes into consideration respondents' perceived actions toward themselves during difficult situations. Each item is rated on a 5-point Likert scale [from “*Almost never” (1)* to “*Almost always” (5)*]. The SCS can be divided into two global dimensions (67, 68): (i) compassionate self-responding: this dimension comprises the “self-kindness” (e.g., “I am kind to myself when I am experiencing suffering”), “common humanity” (e.g., “When I feel inadequate in some way, I try to remind myself that feelings of inadequacy are shared by most people'), and “mindfulness” (e.g., “When I fail at something important to me I try to keep things in perspective”) subscales; (ii) uncompassionate self-responding: comprises the “self-judgement” (e.g., “I am intolerant and impatient toward those aspects of my personality I don't like”), “isolation” (e.g., “When I fail at something that's important to me, I tend to feel alone in my failure”), and “overidentification” (e.g., “When I fail at something important to me, I become consumed by feelings of inadequacy”) subscales. The internal consistency for the SCS was found to be excellent (α = 0.92) in the original study and good in the Portuguese validation study (α = 0.89).

##### Psychological flexibility

Psychological flexibility variable was measured by the *Comprehensive assessment of Acceptance and Commitment Therapy processes* (CompACT) (69, 70), a general self-report measure of the ACT processes which, in its Portuguese version, is composed of 18 items with three dimensions of psychological flexibility: (i) openness to experience (e.g., “I try to stay busy to keep thoughts or feelings from coming”–reverse item), behavioral awareness (e.g., “It seems I am ‘running on automatic' without much awareness of what I'm doing”–reverse item), and valued action (e.g., “I behave in line with my personal values”). Each item is rated on a 7-point scale [from “*Never true” (0)* to “*Always true” (6)*]. Higher scores indicate greater psychological flexibility. In the original study (69), the CompACT presented good internal consistencies (with Cronbach's alphas varying between 0.87 and 0.91). The CompACT's Portuguese 18-item version showed acceptable to good internal consistencies with Cronbach's alphas between 0.77 and 0.88 (70). Also, the CompACT's 18-item was used in another Portuguese study presenting a Cronbach's alpha of 0.84 for the total scale and between 0.76 and 0.88 for the subscales (71).

#### Secondary Outcomes

##### Functional impairment

This outcome was measured by the *Work and Social Adjustment Scale* (WSAS) (72), a 5-item measure of perceived functional impairment in daily activities, such as work, family, interpersonal relations, social and private leisure activities, and home management. Each item is rated on a 9-point scale [“*Not at all” (0)* to “*Very severely” (8)*]. Higher scores denote higher functional impairment. The original study showed good internal consistencies throughout several different conditions (ranging between 0.70 and 0.94). The WSAS's psychometric properties, validity and sensitivity to change have been demonstrated by a wide range of studies (73). In a Portuguese study, the WSAS has shown a good internal consistency (α = 0.87) (55).

##### General quality of life

This outcome was evaluated by the *EUROHIS-QOL 8-item index* (74–76), a quality of life measure composed of eight items (regarding general health, energy, daily living activity, overall quality of life, finances, social relationships, self-esteem, and home) which were extracted from the WHOQOL-Bref. Each item has an individualized 5-point Likert scale (the same response scales used in the WHOQOL-bref). Higher scores indicate higher quality of life. The EUROHIS-QOL 8-item index showed good discriminant validity, and it is considered a useful instrument to evaluate treatment effectiveness (77). The initial study was conducted in 10 countries and revealed an overall Cronbach's alpha of 0.83 (75). Similarly, the Portuguese validation study also showed a good internal consistency (α = 0.83) (76).

##### Health-Related quality of life

This outcome was measured by the *Inflammatory Bowel Disease Questionnaire–UK version* (IBDQ-UK) (78), which is the Anglicized 30-item version of the IBDQ (79, 80), an IBD-specific quality of life instrument. The IBDQ-UK's authors have modified the wording of some questions and simplified their response options. Each item is rated on a 4-point scale (three questions have an additional “non-applicable” option available) ranging from “*no, no at all/none” (0)* to “*on 8 to 14 days (i.e., more than every other day)/Yes, all of the time” (4)*. Participants are asked to answer questions regarding their IBD and how it has affected their lives during the previous 2 weeks. The internal consistency for the IBDQ-UK in its original study was found to be excellent (Cronbach's alpha of 0.94).

##### Chronic illness-related shame

Illness shame was assessed by the *Chronic Illness-related Shame Scale* (CISS) (81), a 7-item unidimensional scale that was specifically designed to evaluate shame associated with the experience of having a chronic illness and/or its related symptoms (e.g., “I feel inadequate because of my illness and symptoms,” “I'm ashamed of talking with others about my illness or symptoms,” “I feel that my illness is embarrassing”). Each item is rated on a 5-point Likert scale [from “*Never true” (0)* to “*Always true” (4)*]. Higher scores reflect higher levels of chronic illness shame. The original study was conducted in Portugal, in which the CISS revealed a very good internal consistency (α = 0.91).

##### IBD symptom perception

Symptom perception was measured by the *IBD symptoms scale* (15), a 16-item self-report Portuguese scale which was developed to evaluate the frequency of IBD symptoms during the precedent month (e.g., fatigue, abdominal pain and bloating, flatulence, diarrhea, nausea or vomiting, fever, the urgency to evacuate). Each item is rated on a 7-point scale [ranging from “*Never” (0)* to “*Always” (6)*]. Higher scores indicate a higher level of IBD symptom perception.

##### Disease activity

The Mayo Score (non-invasive 9-point partial Mayo Score; for ulcerative colitis participants) (82) and Harvey-Bradshaw Score (83) (for Crohn's Disease participants) were analyzed considering assessments by participants and their physician. The physician was blinded to participants' allocation. These scores indicate IBD activity. The partial Mayo score refers to stool frequency, rectal bleeding, and the physician's global assessment; the Harvey Bradshaw Score assesses general well-being, abdominal pain, number of liquid/sift stools, abdominal mass, and presence of extra-intestinal manifestations.

##### Biomarkers

An erythrogram, leukogram, and thrombogram were obtained from all participants since hematological alterations such as anemia, thrombocytosis and leukocytosis, may be observed in some patients, in particular during flares. C Reactive Protein (CRP) was also determined (by immunoturbidimetry) since it is an acute-phase reactant and therefore is a reliable biomarker to assess inflammatory states. Hence, although not specific for IBD, CRP is regularly monitored in patients in order to assess the degree of intestinal inflammation. Finally, serum albumin was determined (by colorimetry) since it tends to decrease rather quickly in severely ill patients. In fact, hypoalbuminemia is frequently observed in patients with uncontrolled disease or with prolonged and frequent flares (84). The blood specimens were analyzed according to the Coulter principle (impedance).

In order to assess a specific biomarker, intestinal inflammation was investigated by quantifying fecal calprotectin. Indeed, fecal calprotectin is a valuable and sensible tool to monitor IBD progression and anticipate relapses, correlating well with the degree of endoscopic and histological inflammation (85). Fecal calprotectin was determined using Buhlmann's Quantum Blue® fcal assay. Briefly, calprotectin was extracted from stools using a Calex® cap pre-filled with extraction buffer and applied to a lateral flow test cassette adsorbed with a monoclonal antibody against human calprotectin. The cassette was then inserted into Buhlmann Quantum Blue® reader which immediately started the reading process, converting the image analysis into a quantitative result (μg/g).

Gut microbiome was sequenced in the stools of patients before and after the psychological intervention in an attempt to establish a mechanist link between bacteria richness and diversity with disease progression (in terms of clinical and psychological outcomes) and biochemical indicators.

### Acceptability Assessment

Participants in the experimental condition answered a questionnaire regarding the quality of the LIFEwithIBD intervention, the provided resources (audio files, participants manual), and participants' perceived change (e.g., IBD symptoms, ability to self-regulate emotions, quality of life, perception of change by closed ones). The intervention's attrition will also be considered a measure of acceptability.

### Data Analysis

All analyses are planned as intent-to-treat (ITT). The primary outcome will be the psychological distress (DASS-21–*depression, anxiety, and stress*) measured at 8 weeks (immediately post-intervention) between the two intervention groups and adjusting for baseline scores using ANCOVA. We will examine the ANCOVA's assumptions through standard tests and residual plots and perform transformations. The magnitude of the differences between-treatment groups will be expressed as the standardized mean difference.

#### Secondary Analyses

Between-groups differences at 8 weeks will be examined for the following continuous outcomes: IBD symptom perception (IBDSS); psychological processes (SCS, CompACT); chronic illness-related shame (CISS); work and social adjustment (WSAS); quality of life (EUROHIS-QOL-8, UK-IBDQ), and disease activity (Mayo Score for ulcerative colitis participants; and Harvey-Bradshaw Score for Crohn's Disease participants). All analyses are to be conducted using ANCOVA with baseline values as a covariate.

#### Longitudinal Outcomes

We will use Mixed Models of Repeated Measures (MMRM) to examine for Group^*^Time interaction effects on each of the continuous outcomes from baseline, 9 weeks, 3 and 12 months; the DASS-21, the IBD symptom perception scale; SCS, CompACT, CISS, WSAS, EUROHIS-QOL-8, UK-IBDQ, and Mayo or Harvey-Bradshaw score.

#### Exploratory Outcomes

Reliable change indices between baseline and 9 weeks will be calculated for all outcomes and correlated with demographics to examine for subgroup efficacy predictors. For exploratory analyses focused upon proportionate data, such as comparisons of mood dysfunction outcomes, McNemars' test can be used to examine within-group differences across time points.

#### Attrition, Protocol Non-adherence, and Missing Data

All participants recruited into the study, regardless of treatment group, are to have as much data as possible recorded as fully as possible in accordance with this protocol. Missing data will be monitored to avoid bias by attrition or patterns of missingness. All cases of missing data, including non-retention of participants or withdrawal of consent, are to be recorded by research staff. Where data is missing, the frequency and reasons for missing data are to be recorded. Missing data is to be examined to see if the missingness is informative to the study, for example, if specific variables are missed systematically by an identifiable participant pattern.

In situations where data is missing, the data collected up to that point will be used provided the participant has not withdrawn their consent or requested that the information be removed. If missing data is considered Missing Completely at Random or Missing at Random (MCAR or MAR), multiple imputation methods will be used to estimate missing values. The accepted minimum number of imputations is five to estimate the missing data from existing data. If data appears to be missing in a non-random way (Missing Not At Random, MNAR), then this data can be examined for potential biases or patterns statistically, and missing values can then be estimated using monotonic methods.

#### Biomarkers

Differences of each biomarker (complete bold count, C reactive protein, plasma albumin and fecal calprotectin will be analyzed intra-individually between T0 and T3 to ascertain the evolution trend for each subject. Then, participants will be divided into three different subpopulations: low-profile (those who are below the reference interval for a given analyte), median-profile (those who fall within the reference interval) and high-profile (those who are above the reference interval). The percentage of individuals who change from the initial (at T0) to another profile at the end of 12 months (T3) will be determined.

Gut microbiota was also analyzed at T0 and T3. Faces were collected at both time points, and total DNA was extracted. The V3–V4 region of 16S rDNA gene was amplified by Polymerase Chain Reaction (PCR), and the amplicons will be pooled at equimolar concentrations and sequenced using high throughput techniques. GelComparII (Applied Maths, Sint-Martens-Latem, Belgium) will be used to analyze and compare bacteria profiles. Cluster analysis will be performed using the UPGMA method (group average method), applying Pearson correlation coefficient. Diversity (Shannon–H') index of bacterial communities will be calculated using PAleontological STatistics (PAST) version 1.34. Significance of differences among diversity index will be performed using the *t*-test through PAST.

## Discussion

Among those designed for or tested in IBD, the LIFEwithIBD intervention is the first to integrate ACT and compassion approaches. The RCT presented in this protocol paper will provide evidence on the efficacy of this intervention (which was delivered along with TAU) in improving health outcomes in comparison to the exclusive delivery of TAU (medical treatment). Due to the inactive nature of the control condition, future studies should compare the intervention's efficacy to other conditions such as active controls (e.g., psychoeducation) and other psychological interventions tested in IBD, such as Cognitive and Behavioral Therapy (86) or Hypnotherapy (87). The use of an inactive control will indeed be a significant limitation to this RCT, especially considering that participants allocated to this condition were aware of their non-allocation to the intervention group, which may influence answers to the administered self-report questionnaires. Also, contact with a group of people with the same pathology, as well as with the therapists, may in itself provide therapeutic effects only experienced by the experimental group, which adds to the disparity between the study groups. Nevertheless, considering this is the first time the LIFEwithIBD intervention will be tested, the use of an inactive control may provide useful preliminary results on the effects of the intervention.

The greatest limitation to this RCT appears to be participant retention, similarly to previous RCTs testing group interventions in this population, which also showed large attrition rates (25, 86). In the current study this may have been due to participants not being compensated for their participation besides receiving the intervention. Results from this RCT will act as support for future studies testing the LIFEwithIBD intervention or other acceptance and/or compassion-based interventions for IBD.

## Ethics Statement

This study was reviewed and approved by Coimbra Hospital and Universitary Center (CHUC), Coimbra, Portugal. Patients/participants provided their written informed consent.

## Author Contributions

CF and IT designed and wrote the project's proposal, obtained funding, and provided study oversight. IT and JP wrote the first draft of the manuscript. DS and BR adapted the project proposal's statistical analysis to this manuscript. All authors provided feedback on the study's design and on this manuscript, and approved its final version.

## Conflict of Interest

Outside the current work, IT received consultancy fees from Pfizer Inc. The remaining authors declare that the research was conducted in the absence of any commercial or financial relationships that could be construed as a potential conflict of interest.

## Publisher's Note

All claims expressed in this article are solely those of the authors and do not necessarily represent those of their affiliated organizations, or those of the publisher, the editors and the reviewers. Any product that may be evaluated in this article, or claim that may be made by its manufacturer, is not guaranteed or endorsed by the publisher.

## Footnotes

^1^Diagnosed disorders were indicated by the physician or the participant; severe depression corresponds to a score ≥20 on the PHQ-9; suicidal ideation was considered present after a positive answer to PHQ-9's last item and subsequent confirming questioning.
