# Corrigendum: The LIFEwithIBD Intervention: Study Protocol for a Randomized Controlled Trial of a Face-to-Face Acceptance and Commitment Therapy and Compassion-Based Intervention Tailored to People With Inflammatory Bowel Disease

**DOI:** 10.3389/fpsyt.2022.837357

**Published:** 2022-02-14

**Authors:** Inês A. Trindade, Joana Pereira, Ana Galhardo, Nuno B. Ferreira, Paola Lucena-Santos, Sérgio A. Carvalho, Sara Oliveira, David Skvarc, Bárbara S. Rocha, Francisco Portela, Cláudia Ferreira

**Affiliations:** ^1^Faculty of Psychology and Education Sciences, CINEICC, University of Coimbra, Coimbra, Portugal; ^2^Department of Molecular and Clinical Medicine, Institute of Medicine, University of Gothenburg, Sahlgrenska Academy, Gothenburg, Sweden; ^3^Instituto Superior Miguel Torga, Coimbra, Portugal; ^4^School of Social Sciences, University of Nicosia, Nicosia, Cyprus; ^5^Universidade Lusófona de Humanidades e Tecnologias, Escola de Psicologia e Ciências da Vida, HEI-Lab, Lisbon, Portugal; ^6^School of Psychology, Deakin University, Geelong, VIC, Australia; ^7^Faculty of Pharmacy, University of Coimbra, Coimbra, Portugal; ^8^Gastroenterology Service, Centro Hospitalar e Universitário de Coimbra (CHUC), Coimbra, Portugal

**Keywords:** acceptance and commitment therapy, compassion, inflammatory bowel disease, mindfulness, randomized controlled trial, study protocol

In the original article, Figure 1 was not correctly presented. The participant flow was not correctly calculated and reported, in particular to what concerned the number of participants from the experimental group with complete baseline assessments. This mistake was identified in the beginning of the conduction of the RCT's statistical analyses, after the present study protocol was published. The flow diagram has been updated and re-designed according to CONSORT 2010 guidelines. The corrected Figure 1 appears below.

**Figure 1:**
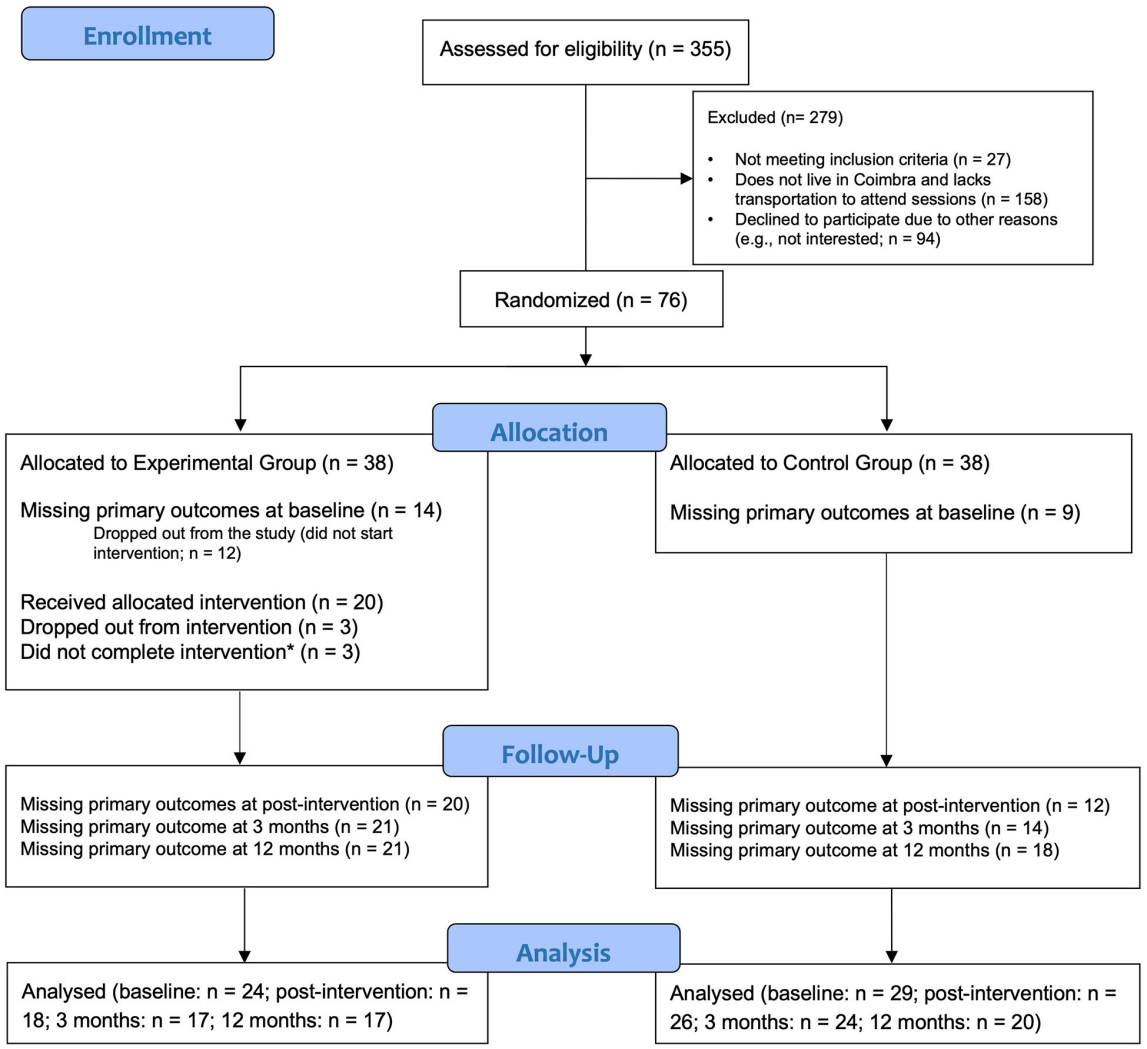
CONSORT 2010 flow diagram. *Participants with three consecutive absences or who attended less than two-thirds of the intervention were considered non-completers.

In the paragraph “Randomization of Participants”, the information provided in the last sentence was incomplete. As originally reported, before the start of the intervention, six patients self-excluded from the study due to a lack of resources to attend the sessions (e.g., lack of time, transportation). In addition to these participants, other 6 participants also dropped out from the study without providing a reason. A correction has been made to this paragraph:

**Randomization of Participants**

Figure 1 shows the flow of participants through the study. Of the 355 patients screened, 279 were excluded for not meeting criteria to participate. Seventy-six participants were randomly assigned to one of two conditions: experimental group (Treatment as Usual + LIFEwithIBD intervention: *n* = 38) or control group (TAU: *n* = 38), through computed-based randomization (www.random.org/lists/). Before the start of the intervention, twelve participants self-excluded from the study; half provided reasons for dropping out, such as lack of time, lack of transportation, or unavailability due to overlapping schedules.

The authors apologize for this error and state that this does not change the scientific conclusions of the article in any way. The original article has been updated.

## Publisher's Note

All claims expressed in this article are solely those of the authors and do not necessarily represent those of their affiliated organizations, or those of the publisher, the editors and the reviewers. Any product that may be evaluated in this article, or claim that may be made by its manufacturer, is not guaranteed or endorsed by the publisher.

