# Corrigendum: The LIFEwithIBD Intervention: Study Protocol for a Randomized Controlled Trial of a Face-to-Face Acceptance and Commitment Therapy and Compassion-Based Intervention Tailored to People With Inflammatory Bowel Disease

**DOI:** 10.3389/fpsyt.2022.969795

**Published:** 2022-07-05

**Authors:** Inês A. Trindade, Joana Pereira, Ana Galhardo, Nuno B. Ferreira, Paola Lucena-Santos, Sérgio A. Carvalho, Sara Oliveira, David Skvarc, Bárbara S. Rocha, Francisco Portela, Cláudia Ferreira

**Affiliations:** ^1^Faculty of Psychology and Education Sciences, CINEICC, University of Coimbra, Coimbra, Portugal; ^2^Department of Molecular and Clinical Medicine, Institute of Medicine, University of Gothenburg, Sahlgrenska Academy, Gothenburg, Sweden; ^3^Instituto Superior Miguel Torga, Coimbra, Portugal; ^4^School of Social Sciences, University of Nicosia, Nicosia, Cyprus; ^5^Universidade Lusófona de Humanidades e Tecnologias, Escola de Psicologia e Ciências da Vida, HEI-Lab, Lisbon, Portugal; ^6^School of Psychology, Deakin University, Geelong, VIC, Australia; ^7^Faculty of Pharmacy, University of Coimbra, Coimbra, Portugal; ^8^Gastroenterology Service, Centro Hospitalar e Universitário de Coimbra (CHUC), Coimbra, Portugal

**Keywords:** acceptance and commitment therapy, compassion, inflammatory bowel disease, mindfulness, randomized controlled trial, study protocol

In the published article, the Funding statement was not sufficiently detailed. This project was peer-reviewed in the context of the 2017 R&D projects Grant call of the Portuguese Foundation for Science and Technology (Fundação para a Ciência e Tecnologia), having successfully been granted funding (Grant No. PTDC/PSI-ESP/28602/2017).

The correct Funding statement appears below.

## Funding

This project (CENTRO-01-0145-FEDER-028602, PTDC/PSI-ESP/28602/2017) was funded by the Fundo Europeu de Desenvolvimento Regional (FEDER) of the European Union, through the Programa Operacional Regional do Centro (CENTRO 2020) of Portugal-2020 and by the Fundação para a Ciência e Tecnologia I.P./MCTES through national funds (PIDDAC).

The authors apologize for this error and state that this does not change the scientific conclusions of the article in any way. The original article has been updated.

## Publisher's Note

All claims expressed in this article are solely those of the authors and do not necessarily represent those of their affiliated organizations, or those of the publisher, the editors and the reviewers. Any product that may be evaluated in this article, or claim that may be made by its manufacturer, is not guaranteed or endorsed by the publisher.

